# Continuous glucose monitoring reveals glycemic profile in adrenal insufficiency patients on glucocorticoid replacement

**DOI:** 10.1530/EC-26-0304

**Published:** 2026-10-26

**Authors:** Cuili Ning, Rui Zhang, Wei Liu, Xianghai Zhou, Yunyang Sun, Xueyao Han, Linong Ji, Qian Ren

**Affiliations:** ^1^ Department of Endocrinology, Shijiazhuang Second Hospital, Shijiazhuang, China; ^2^ Department of Endocrinology, Peking University People’s Hospital, Beijing, China; ^3^ Peking University Diabetes Center, Beijing, China; ^4^ Shanghai iMedpower Tech.LTD, Beijing, China

**Keywords:** adrenal insufficiency, glucocorticoid replacement therapy, continuous glucose monitoring, glucose fluctuation, immune checkpoint inhibitor

## Abstract

**Objective:**

Hypoglycemia is common in patients with adrenal insufficiency (AI), who require lifelong glucocorticoid replacement therapy. However, whether this therapy restores physiological glycemic fluctuations in patients with AI remains unclear. This study aimed to characterize glucose fluctuation pattern in patients with AI using continuous glucose monitoring (CGM).

**Methods:**

Twenty-seven patients with AI on stable doses of glucocorticoid were consecutively enrolled. Fifty-four age-, sex-, and BMI-matched normal controls (NCs) were selected from a healthy volunteer database at a 2:1 ratio. CGM was conducted with a blinded method for 14 days. Glucocorticoid regimen and adverse events during CGM were documented.

**Results:**

The AI group had a mean age of 59.53 ± 8.51 years (15 males). Compared with NCs, patients with AI exhibited increased time below range (TBR, glucose <3.9 and <3.0 mmol/L) and decreased time in range (TIR, glucose within 3.9–7.8 mmol/L). Time above range (TAR, glucose ≥ 7.8 mmol/L) was elevated during the lunch and dinner periods, and glucose fluctuation parameters were also increased. CGM-detected hypoglycemia was observed in all patients (27/27, 100%) versus 46/54 (85.2%) controls (*P* = 0.047). Patients receiving once-daily glucocorticoid had higher lunchtime TAR than those on twice-daily dosing. Hydrocortisone-treated patients showed higher breakfast and lunchtime TAR compared with prednisone users. Patients with immune checkpoint inhibitor (ICI)-induced AI had higher glucose levels and fluctuation parameters during breakfast and dinner periods than those with other etiologies.

**Conclusion:**

Patients with AI receiving glucocorticoid replacement experience more frequent CGM-recorded asymptomatic hypoglycemia and greater glucose variability. CGM may provide valuable insights for optimizing glucocorticoid administration strategies in AI.

## Introduction

The global incidence of primary adrenal insufficiency (PAI) ranges from 93 to 140 cases per million population, while secondary adrenal insufficiency (SAI) affects 150–280 per million individuals (1). Glucocorticoid-induced AI is the most prevalent subtype due to the widespread clinical use of glucocorticoids for various diseases (2). With the increasing use of immune checkpoint inhibitors (ICIs) in oncology, the incidence of ICI-induced AI has risen rapidly in recent years (3). Glucocorticoid replacement has been the standard of care for AI for over 50 years (4). Inadequate dosing may trigger clinical symptoms or even life-threatening adrenal crisis (5), while excessive dosing can lead to Cushing’s syndrome, impaired glucose metabolism or glucocorticoid-induced diabetes (6), osteoporosis, and increased risk of cardiovascular diseases (7), ultimately elevating mortality and morbidity (8). Conversely, inadequate replacement increases the risk of hypoglycemia, especially asymptomatic nocturnal hypoglycemia, which is also associated with higher cardiovascular disease and mortality risk (9). Currently, no reliable objective biomarker is available to guide individualized replacement regimen. Continuous glucose monitoring (CGM) devices enable comprehensive evaluation of glucose profiles and fluctuation trends throughout the day and across days (10). A previous study using CGM identified disrupted glucose homeostasis in patients with hypopituitarism and an association between hyperglycemia and prednisone dosage (11). Another CGM study demonstrated nocturnal hypoglycemia in patients with AI and its resolution following glucocorticoid treatment (12). Prospective controlled trials and cohort studies investigating patients with AI during and outside Ramadan fasting also confirmed the high prevalence of CGM-detected hypoglycemia in this population (13, 14). These previous studies suggested the potential value of CGM in investigating glucose profile in patients with AI. Thus, the main objectives of this study were i) to characterize glucose profiles using CGM in patients with AI with different etiologies compared with age-, sex-, and body mass index (BMI)-matched healthy controls and ii) to explore the association between glucose profiles and glucocorticoid replacement regimens (including type, dosage, and frequency) in patients with AI.

## Materials and methods

### Study design and subjects

This cross-sectional study was conducted in the Department of Endocrinology of our hospital from February to November 2025. A total of 27 consecutive patients with AI from both outpatients and inpatients were enrolled as the AI group. The inclusion criteria were as follows: i) confirmed diagnosis of AI (PAI or SAI) and ii) a stable dose of glucocorticoid therapy for at least 1 month. The exclusion criteria were the following: i) previous diagnosis of diabetes mellitus or use of antidiabetic drugs; ii) myocardial infarction, acute infection, recent surgery, or severe trauma within 3 months; iii) anemia, severe liver disease, or undergoing dialysis; iv) endogenous hyperinsulinemia such as insulinoma, functional beta-cell disorder, or autoimmune insulin hypoglycemia; v) growth hormone deficiency; vi) severe malnutrition; vii) uncontrolled thyroid disease; and viii) any other disorders known to induce spontaneous hypoglycemia.

A total of 54 normal controls (NCs) without known glucometabolic disorders were selected from a CGM database of healthy volunteers enrolled between November 2022 and March 2025. The matching criteria were as follows: i) 2:1 matching (two NCs per AI patient); ii) same sex and age ± 5 years; iii) same BMI categories (BMI < 24 kg/m^2^, 24–28 kg/m^2^, and >28 kg/m^2^). All participants provided written informed consent.

### Clinical data collection

Demographic and clinical data (age, sex, AI duration, glucocorticoid type, and daily dosage) were collected via investigator-administered questionnaires. Hydrocortisone (HC) doses were converted to prednisone (Pred) equivalents using a 4:1 ratio (20 mg hydrocortisone = 5 mg prednisone) based on relative glucocorticoid potencies (15). Height, weight, blood pressure, and heart rate were measured after enrollment. Laboratory parameters included 08:00 h plasma adrenocorticotropic hormone (ACTH) and serum cortisol, electrolytes, blood glucose, lipid profiles, hepatic and renal function markers, and complete blood count.

### CGM data collection

Both AI and NC groups underwent 14-day blinded CGM. The Abbott FreeStyle Libre 1 system (FreeStyle Libre, Abbott, UK) was used for patients with AI, and the SiJoy GS1 system (SiBionics, China) was used for NCs. Both devices measure glucose concentrations in subcutaneous interstitial fluid, are factory-calibrated, require no capillary glucose calibration, are applied to the posterior upper arm, and have a maximum sensor wear duration of 14 days. For FreeStyle Libre 1, the 20/20% consistency was 86%, the percentages of A + B zones of the Clarke and Consensus grid were 99.10 and 99.90%, respectively, and the mean absolute relative difference (MARD) was 10.7%. For SiJoy GS1, the 20/20% consistency was 91.82%, the percentages of A + B zones of the Clarke and Consensus grid were 99.22 and 99.90%, respectively, and the MARD was 8.83 ± 4.03% (16, 17). Both devices satisfy global accuracy standards for CGM.

CGM parameters were defined as follows: time in range (TIR, percentage of time glucose spent within 3.9–7.8 mmol/L), time below range (TBR, percentage of time spent with glucose <3.9 mmol/L and <3.0 mmol/L), and time above range (TAR, percentage of time spent with glucose ≥ 7.8 mmol/L). Glucose fluctuation parameters included standard deviation (SD) of mean glucose, coefficient of variability (CV), mean amplitude of glycemic excursions (MAGE), and mean of daily differences (MODD) of blood glucose. CGM data were analyzed across multiple periods, including 24 h (00:00 am–11:59 pm), diurnal (06:00 am–11:59 pm), and nocturnal (00:00 am–06:00 am) periods, as well as breakfast (08:00 am–10:00 am), lunch (12:00 pm–14:00 pm), and dinner (18:00 pm–20:00 pm) periods.

CGM-detected hypoglycemia was defined as interstitial glucose < 3.9 mmol/L sustained for no less than 15 consecutive minutes. Level 1 hypoglycemia referred to glucose values ranging from 3.0 to 3.9 mmol/L lasting ≥ 15 consecutive minutes. Level 2 hypoglycemia was defined as glucose < 3.0 mmol/L maintained for at least 15 consecutive minutes. Extended hypoglycemia was defined as glucose < 3.9 mmol/L persisting for more than 120 consecutive minutes. The event is considered resolved when interstitial glucose levels return to above the threshold for at least 15 min (10).

### Statistical analysis

Continuous variables with normal distribution are expressed as mean ± standard deviation, and those with skew distribution are presented as median (interquartile range). Between-group comparisons of normally distributed variables were performed using Student’s *t*-test or analysis of variance (ANOVA), and skewed variables were compared using the Mann–Whitney U test or the Kruskal–Wallis H test. Categorical variables were compared using the chi-square test. Statistical significance was set at *P* < 0.05. All tests were two-tailed. Data analysis was performed using SPSS 27.0, and graphs were generated using GraphPad Prism and GPlus Pro.

## Results

### Clinical feature of study population

Clinical characteristics of the 27 patients are shown in Table 1. The mean age was 59.53 ± 8.51 years, with 15 males (55.6%). Only one patient had PAI. The remaining 26 had SAI, including 11 with ICI-induced SAI and 15 with hypopituitarism. The underlying etiologies of hypopituitarism were as follows: empty sella turcica (*n* = 1), Sheehan’s syndrome (*n* = 1), prior pituitary adenoma surgery (*n* = 6), hypophysitis (*n* = 2), pituitary cyst (*n* = 1), iatrogenic secondary AI (*n* = 1), and lung cancer metastasis to the pituitary (*n* = 1). Among them, 21 patients were treated with Pred and 6 with HC. The median daily dosage of glucocorticoids was 5.00 (5.00, 7.50) mg converted to Pred equivalents. The NC group had a mean age of 60.06 ± 8.34 years, including 30 males, and a median BMI of 22.73 (20.53, 26.17) kg/m^2^.

**Table 1:** Clinical feature and laboratory data of the participants.

	AI	NC	*P* value
*n*	27	54	
Male, *n* (%)	15 (55.6)	30 (55.6)	1.000
Age (years)	59.53 ± 8.51	60.06 ± 8.43	0.948
BMI (kg/m^2^)	21.50 (19.57, 27.29)	22.73 (20.53, 26.17)	0.346
Disease duration (month)	8 (2, 36)	NA	NA
Types of glucocorticoids (*n*)		NA	NA
Pred	21		
HC	6		
Daily glucocorticoid dosage (Pred equivalent, mg)	5.00 (5.00, 7.50)	NA	NA
Cortisol (μg/dL) (08:00 h)	1.40 (0.50, 4.08)	NA	NA

BMI, body mass index; Pred, prednisone; HC, hydrocortisone.

### CGM profile characteristics in patients with adrenal insufficiency

All patients with AI completed 14-day CGM with 100% valid data for analysis. The overall glucose fluctuations were greater in patients with AI than in NCs. Compared with age-, sex-, and BMI-matched NCs (Table 2, Figs 1 and 2), patients with AI had significantly higher TBR (both <3.9 mmol/L and <3.0 mmol/L) across 24 h, diurnal, and nocturnal periods – with the most prominent increase during the nocturnal period. CGM-detected hypoglycemia was detected in all patients with AI (27/27, 100.0%), whereas 46 of 54 (85.2%) NCs experienced at least one such episode (*P* = 0.047). Prolonged hypoglycemia occurred in 16 patients (59.3%) but in only 4 controls (7.4%, *P* < 0.001). Level 2 hypoglycemia was documented in 19 patients (70.4%) compared with 15 controls (27.8%, *P* < 0.001). Prolonged level 2 hypoglycemia was present in 10 patients (37.0%) but in none of the controls (*P* < 0.001; Table 2). Additionally, TAR was significantly higher during lunch and dinner periods. Mean glucose (MG) was decreased during nocturnal and breakfast periods but increased during lunch and dinner periods. Other glucose fluctuation parameters, including CV, SD, MAGE, and MODD, were higher than in NCs across 24 h, diurnal, and nocturnal periods.

**Table 2:** Comparison of glucose parameters of CGM between patients and normal controls.

Parameters	AI (*n* = 27)	NC (*n* = 54)	*P*-value
Mean monitoring days	13.92 (13.79, 13.92)	13.90 (11.75, 14.00)	0.683
Overall hypoglycemia			
Number of patients (%)	27 (100)	46 (85.2)	0.047
Number/patient/day	0.79 (0.43, 2.02)	0.32 (0.08, 0.57)	<0.001
Duration (min)	95.00 (45.00, 154.82)	33.85 (24.58, 53.46)	<0.001
Level 1 hypoglycemia			
Number of patients (%)	27 (100)	46 (85.2)	0.047
Number/patient/day	0.79 (0.36, 2.02)	0.29 (0.08, 0.56)	<0.001
Duration (min)	75.00 (52.50, 132.00)	35.00 (24.58, 50.64)	<0.001
Level 2 hypoglycemia			
Number of patients (%)	19 (70.4)	15 (27.8)	<0.001
Number/patient/day	0.14 (0.00, 0.58)	0.00 (0.00, 0.07)	<0.001
Duration (min)	45.00 (0.00, 76.87)	0.00 (0.00, 21.25)	<0.001
Extended hypoglycemia			
Number of patients (%)	16 (59.3)	4 (7.4)	<0.001
Number/patient/day	0.08 (0.00, 0.80)	0.00 (0.00, 0.00)	<0.001
Extended level 2 hypoglycemia			
Number of patients (%)	10 (37.0)	0 (0.0)	<0.001
Number/patient/day	0.00 (0.00, 0.07)	0.00 (0.00, 0.00)	<0.001
Mean glucose (mmol/L)			
24 h period	5.49 (4.99, 6.29)	5.61 (5.42, 6.08)	0.266
Nocturnal period	4.54 (4.00, 4.88)	5.14 (4.93, 5.37)	<0.001
Diurnal period	5.79 (5.27, 6.74)	5.77 (5.56, 6.26)	0.733
Breakfast period	5.51 (4.59, 6.40)	5.77 (5.27, 6.41)	<0.001
Lunch period	6.66 (5.72, 8.03)	6.15 (5.48, 6.93)	<0.001
Dinner period	6.32 (5.39, 7.49)	5.70 (5.23, 6.44)	<0.001
SD (mmol/L)			
24 h period	1.44 (1.20, 1.79)	1.06 (0.92, 1.44)	<0.001
Nocturnal period	0.42 (0.35, 0.58)	0.42 (0.32, 0.55)	0.741
Diurnal period	1.34 (0.99, 1.79)	1.02 (0.85, 1.42)	<0.01
MAGE (mmol/L)			
24 h period	3.45 (2.82, 4.74)	2.50 (2.13, 3.88)	<0.01
Nocturnal period	1.24 (0.89, 1.58)	0.95 (0.72, 1.32)	0.014
Diurnal period	3.54 (2.59, 4.81)	2.56 (2.11, 4.00)	<0.01
MODD (mmol/L)			
24 h period	0.98 (0.83, 1.23)	0.94 (0.74, 1.15)	0.471
Nocturnal period	0.57 (0.42, 0.66)	0.60 (0.46, 0.75)	0.270
Diurnal period	1.09 (0.88, 1.45)	0.97 (0.79, 1.24)	0.123

CV, coefficient of variability; SD, the standard deviation of mean glucose; MAGE, mean amplitude of glycemic excursions; MODD, mean of daily differences of glucose; 24 h period, 00:00 am–11:59 pm; diurnal, 06:00 am–11:59 pm; nocturnal, 00:00 am–06:00 am; breakfast period, 08:00 am–10:00 am; lunch period, 12:00 pm–14:00 pm; dinner period, 18:00 pm–20:00 pm; AI, adrenal insufficiency; NC, normal controls.

**Figure 1:**
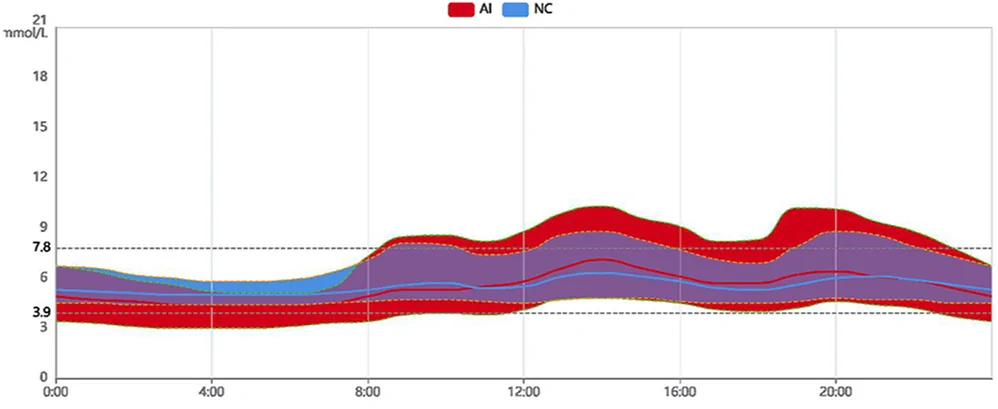
Comparison of ambulatory glucose profiles between AI patient group and normal controls. AI, adrenal insufficiency; NCs, normal controls.

**Figure 2:**
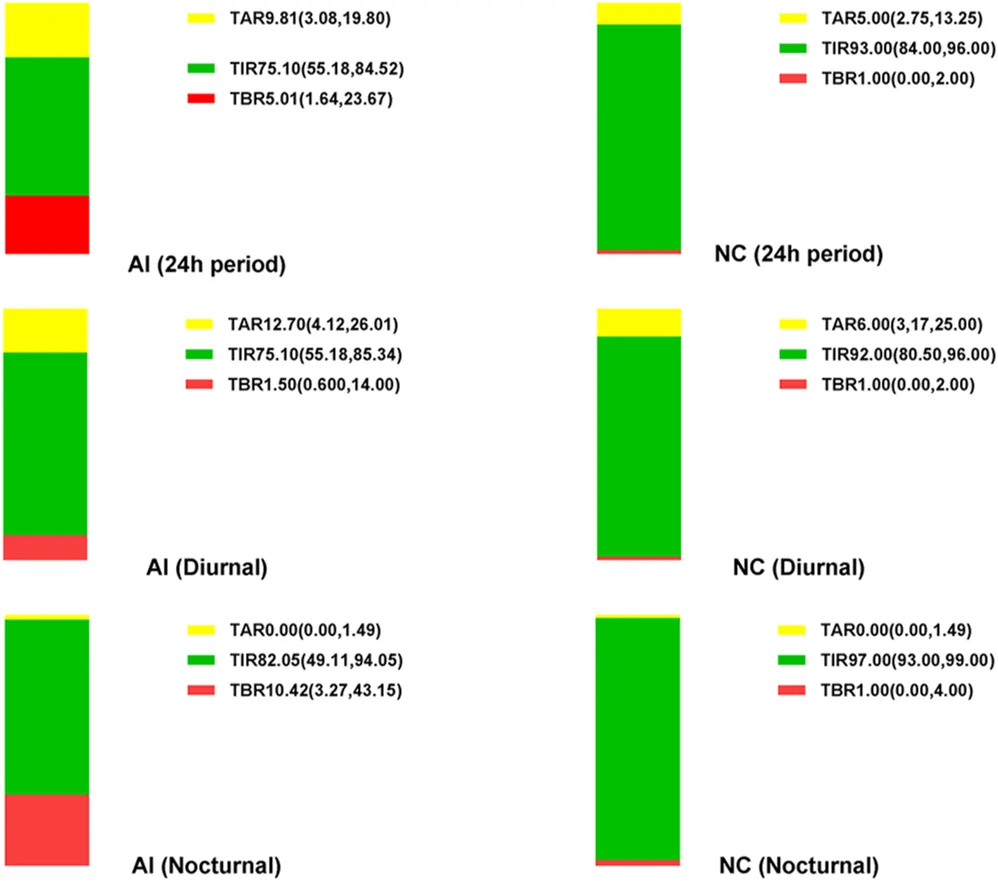
Visualization of TIR, TAR, and TBR in the 24 h, diurnal, and nocturnal periods between AI patient group and normal controls. TBR, glucose < 3.9 mmol/L; TIR, glucose within 3.9–7.8 mmol/L; TAR, glucose ≥ 7.8 mmol/L; 24 h period, 00:00 am–11:59 pm; diurnal, 06:00 am–11:59 pm; nocturnal, 00:00 am–06:00 am; AI, adrenal insufficiency; NC, normal controls.

### Association between CGM profile and glucocorticoid administration

Comparisons of clinical characteristics and glucose parameters by glucocorticoid administration frequency are shown in Supplementary Table 1 (see section on Supplementary materials given at the end of the article) and Fig. 3. No significant differences in age, sex, BMI, or median disease duration were observed between patients receiving once-daily and twice-daily glucocorticoids. However, the once-daily group had a lower median daily glucocorticoid dosage than the twice-daily group. Despite the lower total daily glucocorticoid dosage, the once-daily group exhibited higher lunchtime TAR and greater glycemic variability (SD, MAGE, and MODD) than the twice-daily group. No differences in the prevalence of hypoglycemia, TIR, TBR, or other glucose parameters were found across 24 h, nocturnal, or other meal periods between the two subgroups.

**Figure 3:**
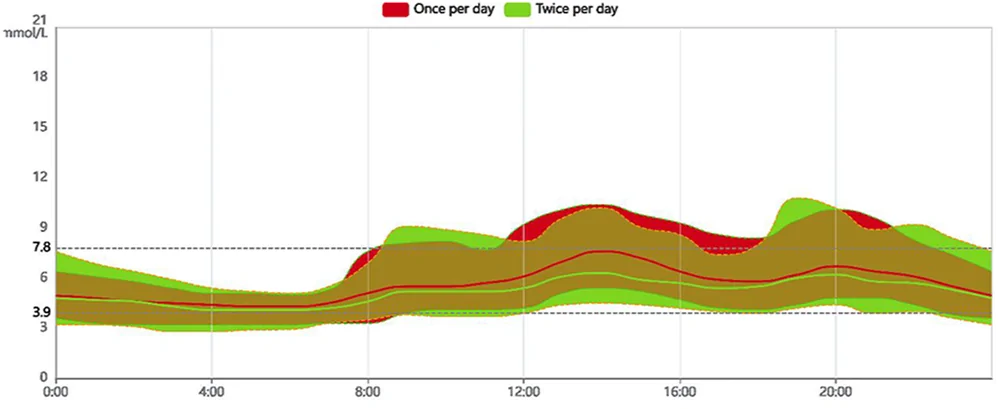
Comparison of ambulatory glucose profiles in patients with AI stratified by glucocorticoid administration frequency.

To explore the association between daily glucocorticoid dosage and glucose profiles, patients were stratified into three subgroups based on Pred-equivalent dosage (5 mg cutoff; Supplementary Table 2). The 5 mg group had a longer disease duration than the <5 mg and >5 mg groups. The <5 mg group had higher nocturnal TIR than the ≥5 mg group. MG and TAR during breakfast and lunch periods were higher in the 5 mg group than in the <5 mg and >5 mg groups.

Comparisons of clinical characteristics and glucose parameters by glucocorticoid type (Pred vs HC) are shown in Supplementary Table 3 and Fig. 4. No significant differences in age, sex, BMI, median disease duration, administration frequency, or daily dosage were observed between the two subgroups. Compared with the Pred group, the HC group had higher MG during nocturnal, breakfast, and lunch periods, as well as higher TAR during breakfast and lunch periods. Additionally, the HC group had higher CV, SD, MAGE, and MODD during the breakfast period.

**Figure 4:**
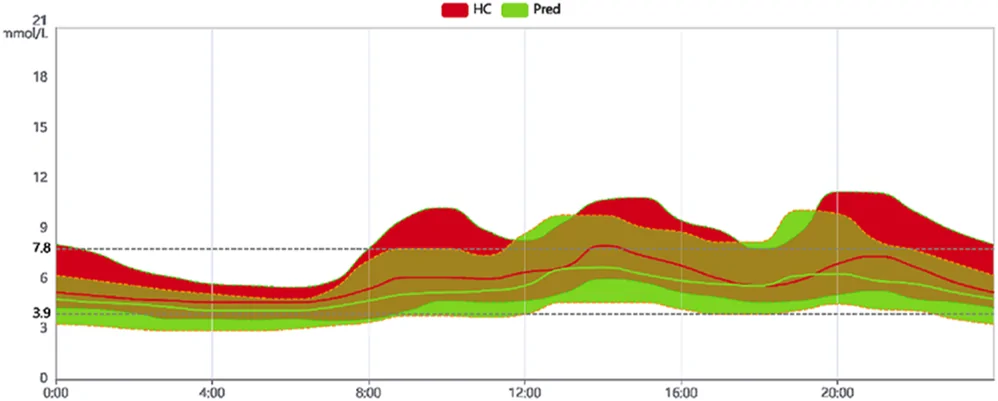
Comparison of ambulatory glucose profiles in patients with AI stratified by glucocorticoid type. Pred, prednisone; HC, hydrocortisone.

### CGM profile characteristics in ICI-induced adrenal insufficiency

Eleven (40.7%) patients had ICI-induced AI. No differences in age, sex, BMI, daily glucocorticoid dosage, or other clinical characteristics were found between patients with ICI-induced AI and those with other etiologies. Compared with the non-ICI-induced group, the ICI-induced AI group had higher MG, TAR, CV, SD, MAGE, and MODD during breakfast and dinner periods (Supplementary Table 4 and Fig. 5).

**Figure 5:**
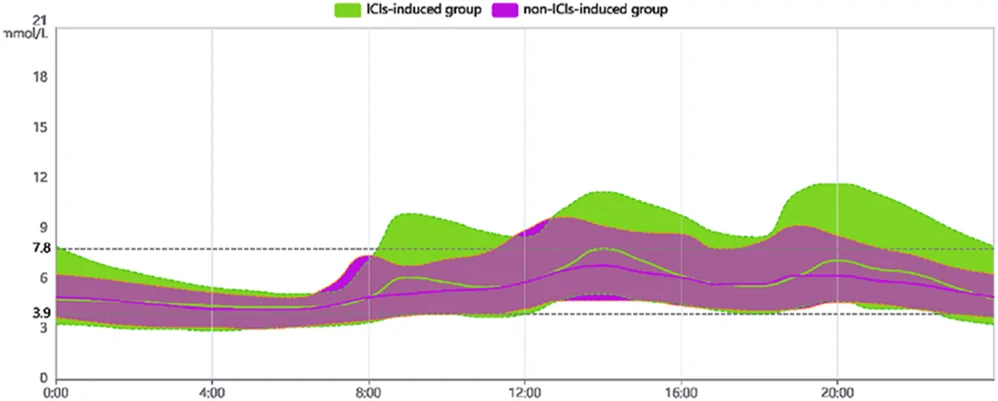
Comparison of ambulatory glucose profiles in patients with AI stratified by glucocorticoid type. Pred, prednisone; HC, hydrocortisone.

### Relationship between adverse events in patient diaries and CGM-detected hypoglycemia

Patients recorded potential hypoglycemic symptoms and other adverse events in diaries. No cardiovascular adverse events were observed during the 14-day CGM monitoring period. Of the 27 patients with CGM-detected hypoglycemia, abnormal symptoms were reported in only 8 patients. Specifically, two cases presented with fatigue, two with fever, and two with gastrointestinal manifestations, including nausea and hyporexia. Additionally, one patient reported nocturnal sleep disturbance, while another developed persistent fatigue and arthralgia. Due to the lack of real-time synchronous symptom documentation, we were unable to determine whether these symptoms coincided with CGM-detected hypoglycemic episodes. Nevertheless, these manifestations were not typical of hypoglycemia-related complaints.

## Discussion

This study demonstrated that patients with AI exhibited an adverse glucose fluctuation profile, characterized by frequent CGM-detected nocturnal hypoglycemia and postprandial hyperglycemia during lunch and dinner periods despite receiving glucocorticoid replacement therapy at physiological dose. Twice-daily glucocorticoid administration was associated with more stable blood glucose levels during the lunch period. Lower glucocorticoid dosage displayed higher nocturnal TIR. Additionally, patients with ICI-induced AI presented with higher glucose levels and greater glycemic variability during breakfast and dinner periods compared with those with AI of other etiologies.

### Hypoglycemia in patients with cortisol deficiency

Consistent with a prior study involving 6 patients with central hypoadrenalism receiving HC replacement therapy, which reported unrecognized nocturnal CGM-detected hypoglycemia in 83% of participants (18), our results showed a 100% occurrence of CGM-detected hypoglycemia in the absence of typical manifestation in patients with AI. Compared with normal controls, these patients also exhibited a substantially higher rate of level 2 and prolonged level 2 hypoglycemia (70.4 and 37.0%, respectively), indicating an elevated risk of hypoglycemia even with stable glucocorticoid replacement. Previous large prospective controlled trials investigating patients with AI during and outside Ramadan fasting further confirmed the high prevalence of CGM-detected hypoglycemia risk in this population under routine glucocorticoid replacement, consistent with our present observations (13, 14). These findings are also broadly consistent with existing CGM evidence from pediatric congenital adrenal insufficiency. One pediatric congenital adrenal hyperplasia (CAH) study reported total monitoring time with glucose<3.9 mmol/L as 9.5% (5.3, 25.5%), while time with glucose below 3.6 mmol/L and 3.0 mmol/L was 9.5% (1.3, 21.0%) and 0.2% (0.1, 1.5%), respectively (19). Another study of children with combined ACTH and GH deficiency showed 1.3% of monitoring time with glucose <2.78 mmol/L and 5% below 3.33 mmol/L (20). Previous research has reported that, unlike pediatric patients, most adult patients with AI develop asymptomatic hypoglycemia, which may only present as nocturnal headaches (12, 21). This is also in line with our findings, as none of the patients in our cohort recorded typical hypoglycemic symptoms in their diaries.

The mechanisms underlying hypoglycemia in patients with AI are related to the physiological actions of cortisol, which is a key gluconeogenic hormone to maintain fasting blood glucose by promoting gluconeogenesis and antagonizing insulin action. Thus, cortisol deficiency in patients with AI predisposes them to hypoglycemia, particularly nocturnal hypoglycemia, which is closely linked to the physiological circadian rhythm of cortisol secretion. Endogenous cortisol secretion typically peaks in the early morning (a phenomenon known as the dawn effect) (22). Cortisol deficiency reduces hepatic glucose production, enhances peripheral glucose utilization, and increases insulin sensitivity (23). Furthermore, cortisol insufficiency severely impairs the body’s counterregulatory response to hypoglycemia by blunting the glucose-elevating effects of glucagon and epinephrine. Thus, despite glucocorticoid replacement, the risks of hypoglycemia and asymptomatic hypoglycemia remain elevated in this population.

### Glucocorticoid replacement and postprandial hyperglycemia

Prior studies have documented an increased prevalence of impaired glucose metabolism, central adiposity, and dyslipidemia in patients with AI (6). Long-term glucocorticoid treatment can induce peripheral insulin resistance by impairing insulin signaling, promote abdominal obesity, and result in hyperglycemia (24). In addition, glucocorticoid administration stimulates glucagon secretion, further perturbing glucose homeostasis and contributing to hyperglycemia (24). Supraphysiological peaks in serum cortisol concentrations typically occur in the daytime following glucocorticoid administration, followed by a trough before the next dose (25). This pharmacokinetic pattern aligns with the observation of increased TAR during lunch and dinner periods in this study.

The combined effects of elevated TBR and postprandial TAR resulted in reduced TIR in patients with AI. Moreover, glycemic variability parameters, including CV, SD, MAGE, and MODD, were also increased in this population. Notably, these CGM-derived parameters have been shown to correlate with adverse cardiovascular outcomes in patients with diabetes (26, 27). Thus, CGM provides a comprehensive and intuitive approach to detecting asymptomatic hypoglycemia and subtle hyperglycemia, which may facilitate the rational adjustment of glucocorticoid dosage and administration timing in patients with AI.

### Impacts of glucocorticoid regimen on glycemic profiles

Patients with AI require lifelong glucocorticoid replacement therapy, and the ideal regimen should mimic the endogenous cortisol rhythm, characterized by a nadir at bedtime and a gradual increase to an early-morning peak just before awakening. HC remains the first-line agent for glucocorticoid replacement therapy in patients with AI (28) and is generally administered two or three times daily (28), whereas Pred is typically given once daily (29). Pediatric endocrine guidelines routinely recommend multiple daily divided doses to mimic physiological circadian cortisol secretion. In contrast, substantial heterogeneity exists in glucocorticoid replacement regimens for adult patients worldwide (30). Emerging formulations, such as once-daily dual-release HC tablets, have been shown to mimic the physiological diurnal cortisol profile (31), which also reducing body weight and glycated hemoglobin (HbA1c) (32). Continuous subcutaneous HC infusion via specialized pumps has also been shown to achieve near-physiological cortisol concentrations (33). However, these novel regimens have not been widely adopted in clinical practice in China. In the absence of robust evidence supporting the superiority of any specific glucocorticoid regimen (5), the selection of glucocorticoid is often determined by a combination of patient factors and clinician judgment. The present study compared the CGM-derived glucose profiles across different glucocorticoid regimens and found that twice-daily dosing was associated with more stable blood glucose levels during lunch periods, suggesting that this administration frequency may approximate the physiological cortisol rhythm, regardless of whether HC or Pred is used. We also found that patients receiving HC treatment had higher TAR during breakfast and lunch periods, as well as greater CV, SD, MAGE, and MODD during breakfast periods compared with Pred users, despite similar median glucocorticoid dosage between the two subgroups. This finding may be attributed to the short half-life of HC, which leads to a rapid elevation in serum cortisol concentration followed by a precipitous decline, resulting in a more pronounced peak–trough fluctuation (34).

Previous studies have shown that a daily Pred dose exceeding 5 mg exerts more deleterious effects on glycometabolism than a dose of ≤ 5 mg in patients with hypopituitarism (11). However, the present study found that patients receiving a daily Pred dose of 5 mg had higher postprandial TAR and MG levels than the < 5 mg and > 5 mg groups. This discrepancy may be explained by the longer disease duration in the 5 mg group as prolonged glucocorticoid replacement therapy may increase peripheral insulin resistance, promote hepatic gluconeogenesis, and elevate postprandial glycemic excursions (30, 35). Nevertheless, the observation that nocturnal TIR was higher in patients with lower dosage still suggests the potential benefits of lower dosage of glucocorticoids. Prior research has demonstrated that patients with AI with a history of exposure to supraphysiological doses of glucocorticoids have an increased risk of mortality (36). Excessive glucocorticoid exposure in patients with AI is associated with adverse clinical outcomes, including elevated BMI, dyslipidemia, decreased bone mineral density, and impaired quality of life (37, 38, 39). The subgroup comparison in the present study constitutes an exploratory post hoc analysis restricted by small sample size and unadjusted confounding factors. Further large-scale studies adjusting for confounding factors are warranted to validate the dose-dependent glycemic phenotypes of glucocorticoid replacement in AI populations and confirm whether CGM-guided personalized dosing improves long-term metabolic outcomes.

### Distinct glycemic features of immune checkpoint inhibitor-induced AI

As an increasingly prevalent novel etiology of AI, ICI-induced AI differs from the conventional AI in terms of underlying mechanisms, clinical characteristics, treatment strategies, and outcomes, many of which remain poorly understood. Our study is the first to investigate the differences in clinical features and glucose pattern between patients with ICI-induced AI and those of other etiologies. Endocrine immune-related adverse events (irAEs) affect up to 40% of patients receiving ICIs, with the incidence varies by the type of ICIs used (40). More complex factors may influence the glucose metabolism in patients with ICI-induced AI, including concurrent antineoplastic medication, and inflammatory effects of tumor immunity may collectively account for the elevated glucose levels observed during breakfast and dinner periods in this subgroup. Future studies are needed to elucidate the key factors regulating glucose metabolism in ICI-induced AI and to develop optimized glucocorticoid replacement regimens tailored to this population.

### Major strengths of the study

A major strength of this study is that it is among the few to employ CGM to characterize glycemic fluctuation patterns in patients with AI. It also revealed associations between glucocorticoid replacement regimens – including drug type, dosage, and administration frequency – and the risk of hypoglycemia or hyperglycemia. Furthermore, our findings suggest that patients with AI with different etiologies may exhibit distinct glucose profiles. Additionally, the strict matching of patients with AI and NCs by age, sex, and BMI effectively minimized confounding variables, enhancing the reliability of our results.

### Limitations and future research directions

This study also has several limitations. First, this is a single-center study with a relatively small sample size. The majority of the included patients had SAI and took Pred. The generalizability of our findings to patients with AI with diverse etiologies is limited. One single primary adrenal insufficiency case was included, introducing minor etiological heterogeneity. We have performed sensitivity analysis excluding this PAI case, and relevant results are given in Supplementary Table 5. Second, different CGM devices were used for patients with AI and NCs. Although both devices have been validated to have acceptable measurement accuracy, inter-device systematic bias may interfere with direct comparison of absolute glucose values between groups. Only the overall trend of glycemic fluctuation profiles is comparable in this study. Meanwhile, complete laboratory test data of healthy controls were not archived in our volunteer database, restricting further intergroup laboratory variable comparison. Third, this study did not include biochemical confirmation or clinical diagnosis of hypoglycemia as a study end point. All low-glucose results are derived from CGM metrics per international consensus definition, which only reflects hypoglycemia risk rather than definitive clinical hypoglycemia. Finally, confounding factors were not fully adjusted in the subgroup analyses of different glucocorticoid regimens, and predefined subgroup analyses could not be performed due to the small sample size. Therefore, multicenter, large-scale prospective studies and randomized controlled trials are needed to confirm whether CGM-guided individualized glucocorticoid replacement regimens can effectively stabilize blood glucose levels, improve quality of life, and enhance long-term prognosis in patients with AI.

## Conclusion

In conclusion, patients with adrenal insufficiency exhibit a distinct glucose fluctuation pattern characterized by asymptomatic CGM-recorded hypoglycemia and prominent postprandial hyperglycemia even under routine glucocorticoid replacement therapy. The application of CGM can provide objective glycemic evidence to facilitate individualized optimization of glucocorticoid replacement regimens in patients with adrenal insufficiency.

## Supplementary materials



## Declaration of interest

The authors declare that there is no conflict of interest that could be perceived as prejudicing the impartiality of the work reported.

## Funding

This work was funded by the Noncommunicable Chronic Diseases-National Science and Technology Major Project (2025ZD0549400, 2025ZD0549401, 2024ZD0532000, and 2024ZD0532005), National Natural Science Foundation of China (82470859), the Beijing Natural Science Foundation (7242157), and the 2024 National Clinical Key Specialty Construction Program of China (Department of Endocrinology, Peking University People’s Hospital) with support from the central government budget.

## Ethics statement

Ethical approval for this study was obtained from the Peking University People’s Hospital Institutional Review Board (Approval No. 2025PHB101-001). All procedures involving human participants were performed in accordance with the ethical standards of the institutional and/or national research committee and with the 1964 Declaration of Helsinki and its later amendments or comparable ethical standards.

## References

[1] Lewis A, Thant AA, Aslam A, et al. Diagnosis and management of adrenal insufficiency. Clin Med 2023 23 115–118. (10.7861/clinmed.2023-0067) PMC11046533 36958832

[2] Beuschlein F, Else T, Bancos I, et al. European Society of Endocrinology and Endocrine Society Joint Clinical Guideline: diagnosis and therapy of glucocorticoid-induced adrenal insufficiency. Eur J Endocrinol 2024 190 G25–G51. (10.1093/ejendo/lvae029) 38714321

[3] Martella S, Lucas M, Porcu M, et al. Primary adrenal insufficiency induced by immune checkpoint inhibitors: biological, clinical, and radiological aspects. Semin Oncol 2023 50 144–148. (10.1053/j.seminoncol.2023.11.003) 38151399

[4] Johannsson G, Falorni A, Skrtic S, et al. Adrenal insufficiency: review of clinical outcomes with current glucocorticoid replacement therapy. Clin Endocrinol 2015 82 2–11. (10.1111/cen.12603) 25187037

[5] Vaidya A, Findling J & Bancos I. Adrenal insufficiency in adults: a review. JAMA 2025 334 714–725. (10.1001/jama.2025.5485) 40522647

[6] Giordano R, Marzotti S, Balbo M, et al. Metabolic and cardiovascular profile in patients with Addison’s disease under conventional glucocorticoid replacement. J Endocrinol Investig 2009 32 917–923. (10.1007/bf03345773) 19620820

[7] Walker BR. Glucocorticoids and cardiovascular disease. Eur J Endocrinol 2007 157 545–559. (10.1530/eje-07-0455) 17984234

[8] Mazziotti G, Formenti AM, Frara S, et al. Management of endocrine disease: risk of overtreatment in patients with adrenal insufficiency: current and emerging aspects. Eur J Endocrinol 2017 177 R231–R248. (10.1530/eje-17-0154) 28583942

[9] Debono M, Ross RJ & Newell-Price J. Inadequacies of glucocorticoid replacement and improvements by physiological circadian therapy. Eur J Endocrinol 2009 160 719–729. (10.1530/eje-08-0874) 19168600

[10] Battelino T, Alexander CM, Amiel SA, et al. Continuous glucose monitoring and metrics for clinical trials: an international consensus statement. Lancet Diabetes Endocrinol 2023 11 42–57. (10.1016/s2213-8587(22)00319-9) 36493795

[11] Han MM, Zhang JX, Liu ZA, et al. Glucose metabolism profile recorded by flash glucose monitoring system in patients with hypopituitarism during prednisone replacement. World J Diabetes 2023 14 1112–1125. (10.4239/wjd.v14.i7.1112) PMC10401453 37547590

[12] Kanazawa K, Hijikata M, Koga S, et al. Impact of glucocorticoid replacement therapy on nocturnal hypoglycemia in adrenal insufficiency: an analysis of multiple case studies. Cureus 2024 16 e61456. (10.7759/cureus.61456) PMC11214865 38947638

[13] Chihaoui M, Sta J, Kamoun E, et al. Hypoglycaemia and other risks of ramadan fasting in patients with primary adrenal insufficiency: a prospective controlled trial using 24-hour glucose monitoring. J Endocrinol Investig 2026 49 359–367. (10.1007/s40618-025-02728-9) 41108503

[14] Chihaoui M, Grira W, Bettaieb J, et al. The risk for hypoglycemia during Ramadan fasting in patients with adrenal insufficiency. Nutrition 2018 45 99–103. (10.1016/j.nut.2017.07.014) 29129244

[15] Beuschlein F, Else T, Bancos I, et al. European Society of Endocrinology and Endocrine Society joint clinical guideline: diagnosis and therapy of glucocorticoid-induced adrenal insufficiency. J Clin Endocrinol Metab 2024 109 1657–1683. (10.1210/clinem/dgae250) PMC11180513 38724043

[16] Ji L, Guo X, Guo L, et al. A multicenter evaluation of the performance and usability of a novel glucose monitoring system in Chinese adults with diabetes. J Diabetes Sci Technol 2017 11 290–295. (10.1177/1932296816662884) PMC5478018 27559031

[17] Yan L, Li Q, Guan Q, et al. Evaluation of the performance and usability of a novel continuous glucose monitoring system. Int J Diabetes Dev Ctries 2023 43 551–558. (10.1007/s13410-022-01112-0)

[18] Watanabe T, Ozawa A, Ishii S, et al. Usage of continuous glucose monitoring (CGM) for detecting an unrecognized hypoglycemia and management of glucocorticoid replacement therapy in adult patients with central hypoadrenalism. Endocr J 2018 65 547–556. (10.1507/endocrj.ej16-0387) 29618670

[19] Galderisi A, Kariyawasam D, Stoupa A, et al. Glucose pattern in children with classical congenital adrenal hyperplasia: evidence from continuous glucose monitoring. Eur J Endocrinol 2023 189 K19–K24. (10.1093/ejendo/lvad147) 37952170

[20] Cambiaso P, Schiaffini R, Pontrelli G, et al. Nocturnal hypoglycaemia in ACTH and GH deficient children: role of continuous glucose monitoring. Clin Endocrinol 2013 79 232–237. (10.1111/cen.12123) 23215896

[21] Kawahara T, Tsuji M, Tominaga N, et al. Frequency of adrenal insufficiency in patients with hypoglycemia in an emergency department: a cross-sectional study. J Endocr Soc 2022 6 bvac119. (10.1210/jendso/bvac119) PMC9419498 36042975

[22] Stalder T, Oster H, Abelson JL, et al. The cortisol awakening response: regulation and functional significance. Endocr Rev 2025 46 43–59. (10.1210/endrev/bnae024) 39177247

[23] Christiansen JJ, Djurhuus CB, Gravholt CH, et al. Effects of cortisol on carbohydrate, lipid, and protein metabolism: studies of acute cortisol withdrawal in adrenocortical failure. J Clin Endocrinol Metab 2007 92 3553–3559. (10.1210/jc.2007-0445) 17609300

[24] Rafacho A, Ortsäter H, Nadal A, et al. Glucocorticoid treatment and endocrine pancreas function: implications for glucose homeostasis, insulin resistance and diabetes. J Endocrinol 2014 223 R49–R62. (10.1530/joe-14-0373) 25271217

[25] Mah PM, Jenkins RC, Rostami-Hodjegan A, et al. Weight-related dosing, timing and monitoring hydrocortisone replacement therapy in patients with adrenal insufficiency. Clin Endocrinol 2004 61 367–375. (10.1111/j.1365-2265.2004.02106.x) 15355454

[26] Reed J, Dong T, Eaton E, et al. Continuous glucose monitoring for glycaemic control and cardiovascular risk reduction in patients with type 2 diabetes not on insulin therapy: a clinical trial. Diabetes Obes Metab 2024 26 2881–2889. (10.1111/dom.15608) 38680050

[27] Cai J, Liu J, Lu J, et al. Impact of time in tight range on all-cause and cardiovascular mortality in type 2 diabetes: a prospective cohort study. Diabetes Obes Metab 2025 27 2154–2162. (10.1111/dom.16212) PMC11885067 39868655

[28] Forss M, Batcheller G, Skrtic S, et al. Current practice of glucocorticoid replacement therapy and patient-perceived health outcomes in adrenal insufficiency – a worldwide patient survey. BMC Endocr Disord 2012 12 8. (10.1186/1472-6823-12-8) PMC3403959 22695167

[29] Sharma A, Lazarus K, Papadopoulou D, et al. Optimising prednisolone or prednisone replacement in adrenal insufficiency. Endocr Connect 2023 12 e230097. (10.1530/ec-23-0097) PMC10388668 37289726

[30] Graziadio C, Hasenmajer V, Venneri MA, et al. Glycometabolic alterations in secondary adrenal insufficiency: does replacement therapy play a role? Front Endocrinol 2018 9 434. (10.3389/fendo.2018.00434) PMC6085438 30123187

[31] Johannsson G, Nilsson AG, Bergthorsdottir R, et al. Improved cortisol exposure-time profile and outcome in patients with adrenal insufficiency: a prospective randomized trial of a novel hydrocortisone dual-release formulation. J Clin Endocrinol Metab 2012 97 473–481. (10.1210/jc.2011-1926) 22112807

[32] Quinkler M, Miodini Nilsen R, Zopf K, et al. Modified-release hydrocortisone decreases BMI and HbA1c in patients with primary and secondary adrenal insufficiency. Eur J Endocrinol 2015 172 619–626. (10.1530/eje-14-1114) 25656494

[33] Oksnes M, Björnsdottir S, Isaksson M, et al. Continuous subcutaneous hydrocortisone infusion versus oral hydrocortisone replacement for treatment of Addison’S disease: a randomized clinical trial. J Clin Endocrinol Metab 2014 99 1665–1674. (10.1210/jc.2013-4253) 24517155

[34] Verma S, Vanryzin C, Sinaii N, et al. A pharmacokinetic and pharmacodynamic study of delayed- and extended-release hydrocortisone (Chronocort) vs. conventional hydrocortisone (Cortef) in the treatment of congenital adrenal hyperplasia. Clin Endocrinol 2010 72 441–447. (10.1111/j.1365-2265.2009.03636.x) PMC2866132 19486026

[35] Guarnotta V, Tomasello L & Giordano C. Prediction of diabetes mellitus induced by steroid overtreatment in adrenal insufficiency. Sci Rep 2022 12 885. (10.1038/s41598-022-04904-w) PMC8766568 35042934

[36] Bergthorsdottir R, Leonsson-Zachrisson M, Odén A, et al. Premature mortality in patients with Addison’s disease: a population-based study. J Clin Endocrinol Metab 2006 91 4849–4853. (10.1210/jc.2006-0076) 16968806

[37] Filipsson H, Monson JP, Koltowska-Häggström M, et al. The impact of glucocorticoid replacement regimens on metabolic outcome and comorbidity in hypopituitary patients. J Clin Endocrinol Metab 2006 91 3954–3961. (10.1210/jc.2006-0524) 16895963

[38] Koetz KR, Ventz M, Diederich S, et al. Bone mineral density is not significantly reduced in adult patients on low-dose glucocorticoid replacement therapy. J Clin Endocrinol Metab 2012 97 85–92. (10.1210/jc.2011-2036) 21994966

[39] Ragnarsson O, Mattsson AF, Monson JP, et al. The relationship between glucocorticoid replacement and quality of life in 2737 hypopituitary patients. Eur J Endocrinol 2014 171 571–579. (10.1530/eje-14-0397) 25127712

[40] Martins F, Sofiya L, Sykiotis GP, et al. Adverse effects of immune-checkpoint inhibitors: epidemiology, management and surveillance. Nat Rev Clin Oncol 2019 16 563–580. (10.1038/s41571-019-0218-0) 31092901

