# Supplementary material for: Continuous glucose monitoring reveals glycemic profile in adrenal insufficiency patients on glucocorticoid replacement
Source: Endocr Connect. 2026 Oct 26;15(10):e260304. doi: 10.1530/EC-26-0304 (PMC13726395; doi:10.1530/EC-26-0304)
Supplement: Supplementary file 1 [file EC-26-0304_supplementary_tables.pdf]

Supplementary Table 1. Comparison of clinical characteristics and CGM parameters in patient group classified by administration frequency.

Parameters		Once daily	Twice daily	p-Value
N		15	12	
Age (yrs)		61.60±8.74	57.83±8.08	0.953
Male, n (%)		6 (40)	9 (75)	0.074
BMI (kg/m^2)		24.05±4.70	21.31±2.82	0.073
Disease duration (month)		30.0(2.0,66.0)	6.0(2.8,10.3)	0.078
Daily glucocorticoid dosage (based on Pred, mg)		5.00(3.75,5.00)	6.88(5.00,7.50)	0.047
Types of glucocorticoids(n)		Pred (12) HC (3)	Pred (9) HC (3)	1.000
Overall hypoglycemia	Number of patients(%)	15(100)	12(100)	1.000
	Number/patient /day	0.76(0.29,2.02)	1.15(0.45,2.23)	0.542
	Duration (min)	78.75(58.13,145.91)	107.45(45.00,160.21)	0.845
Level 1 hypoglycemia	Number of patients(%)	15(100)	12(100)	1.000
	Number/patient /day	0.76(0.29,2.02)	1.15(0.43,2.23)	0.407
	Duration (min)	69.00(61.07,136.61)	94.29(45.63,128.29)	0.732
Level 2	Number of	11(77.3)	8(66.7)	1.000

hypoglycemia	patients(%)			
	Number/patient /day	0.72(0.00,0.58)	0.22(0.00,0.66)	0.824
	Duration (min)	45.00(0.00,63.75)	35.63(0.00,91.72)	0.961
Extended hypoglycemia	Number of patients(%)	9(60.0)	7(58.3)	1.000
	Number/patient /day	0.76(0.00,0.80)	0.29(0.00,0.90)	0.649
Extended Level 2 hypoglycemia	Number of patients(%)	5(33.3)	59(41.7)	0.706
	Number/patient /day	0.00(0.00,0.72)	0.00(0.00,0.72)	0.865
Mean monitoring days		13.92(13.79,13.92)	13.92(13.67,13.92)	0.722
Mean glucose (mmol/L)	24h period	5.80±0.96	5.48±1.26	0.458
	Nocturnal period	4.48±0.65	4.43±1.08	0.885
	Diurnal period	6.17±1.08	5.78±1.34	0.417
	Breakfast period	5.79(4.52,6.49)	5.24(4.62,6.17)	0.163
	Lunch period	7.11(6.00,8.28)	6.25(5.40,7.65)	<0.001
	Dinner period	6.34(5.54,7.37)	6.29(5.00,7.56)	0.312
TIR (3.9-7.8mmol/L) (%)	24h period	71.95±14.15	69.07±21.91	0.831
	Nocturnal period	85.42(55.36,94.05)	75.60(25.01,90.18)	0.305
	Diurnal period	73.99(68.30,82.25)	81.70(48.42,90.50)	0.464

TAR (≥7.8 mmol/L)(%)	24h period	10.53(3.14,20.18)	4.97(1.09,14.54)	0.242
	Nocturnal period	0.00(0.00,1.49)	0.00(0.00,1.20)	0.809
	Diurnal period	14.06(4.19,26.75)	6.60(1.46,19.07)	0.242
	Breakfast period	0.00(0.00,12.50)	0.00(0.00,14.93)	0.730
	Lunch period	33.33(0.00,62.50)	0.00(0.00,42.22)	<0.001
	Dinner period	0.00(0.00,37.50)	0.00(0.00,44.44)	0.361
TBR (<3.9mmol/L) (%)	24h period	3.67(1.58,21.77)	9.41(1.70,27.39)	0.591
	Nocturnal period	10.12(3.27,26.31)	15.77(3.87,69.02)	0.751
	Diurnal period	1.40(0.60,16.87)	4.79(0.55,13.87)	0.643
	Breakfast period	0.00(0.00,11.11)	0.00(0.00,27.78)	0.852
	Lunch period	0.00(0.00,0.00)	0.00(0.00,0.00)	0.365
	Dinner period	0.00(0.00,0.00)	0.00(0.00,0.00)	0.522
TBR (<3mmol/L) (%)	24h period	0.30(0.08,2.85)	0.60(0.02,4.55)	0.959
	Nocturnal period	0.89(0.30,6.55)	2.23(0.00,14.18)	0.961
	Diurnal period	0.20(0.00,1.61)	0.20(0.00,1.65)	0.959
CV (%)	24h period	28.85±5.62	28.24±8.45	0.282
	Nocturnal period	10.19±2.88	10.54±3.49	0.778
	Diurnal period	24.23±4.48	23.88±8.36	0.889
	Breakfast period	12.38(7.27,19.36)	13.70(6.94,19.44)	0.931
	Lunch period	13.88(9.23,19.08)	12.60(8.04,20.80)	0.370
	Dinner period	13.39(8.65,20.66)	12.17(6.41,21.92)	0.175
SD	24h period	1.48(1.37,1.79)	1.24(1.09,2.05)	0.157

(mmol/L)	Nocturnal period	0.48(0.29,0.58)	0.41(0.36,0.58)	0.661
	Diurnal period	1.37(1.19,1.79)	1.13(0.92,1.79)	0.205
	Breakfast period	0.76(0.40,1.09)	0.66(0.33,1.19)	0.626
	Lunch period	0.91(0.64,1.42)	0.82(0.48,1.29)	0.026
	Dinner period	0.84(0.55,1.28)	0.72(0.35,1.38)	0.175
MAGE	24h period	3.59(2.98,4.74)	3.01(2.50,5.23)	0.354
(mmol/L)	Nocturnal period	1.27(0.89,1.63)	1.09(0.89,1.50)	0.591
	Diurnal period	4.08±1.42	3.72±1.73	0.557
	Breakfast period	1.95(1,2.9)	1.70(0.80,3.18)	0.539
	Lunch period	2.40(1.60,3.80)	2.00(1.20,3.50)	0.036
	Dinner period	2.10(1.40,3.20)	1.83(0.89,3.90)	0.293
MODD	24h period	0.99(0.89,1.17)	0.84(0.72,1.26)	0.118
(mmol/L)	Nocturnal period	0.59(0.52,0.66)	0.50(0.34,0.85)	0.464
	Diurnal period	1.12(0.99,1.46)	0.93(0.82,1.43)	0.157
	Breakfast period	0.93(0.53,1.4)	0.69(0.48,1.21)	0.042
	Lunch period	1.34(0.88,2.01)	1.11(0.65,1.91)	0.025
	Dinner period	1.28(0.87,1.92)	0.96(0.61,1.9)	<0.01

BMI, body mass index; Pred, prednisone; HC, hydrocortisone; CV, coefficient of variability; SD, the standard deviation of mean glucose; MAGE, mean amplitude of glycemic excursions; MODD, mean of daily differences of glucose; TBR, time below range, glucose <3.9 mmol/L and <3.0 mmol/L; TIR, time in range, glucose within 3.9-7.8 mmol/L; TAR, time above range, glucose ≥7.8 mmol/L; 24h period, 0:00

am-11:59 pm; Diurnal ,6:00 am to 11:59 pm; Nocturnal, 0:00 am to 6:00 am; Breakfast period, 8:00 am-10:00 am; Lunch period, 12:00 pm-14:00 pm; Dinner period, 18:00 pm-20:00 pm;

Supplementary Table 2. Comparison of clinical characteristics and CGM parameters according to daily prednisolone-equivalent dose of glucocorticoids.

parameters		<5 mg	5 mg	>5 mg	p-Value
N		6	13	8	
Age		55.67±11.31	62.38±8.30	59.13±5.64	0.274
Male, n (%)		0 (0)	9 (69)	6 (75)	<0.01
BMI (kg/m^2)		24.23±4.15	22.63±4.77	22.10±3.14	0.635
Disease duration (month)		11.5(6.3,31.5)	21.0(5.5,75.0)	3.5(1.3,5.8)	0.020
Daily glucocorticoid dosage (based on Pred, mg)		2.50(2.19,3.75)	5.00(5.00,5.00)	7.50(7.50,9.38)	<0.001
Types of glucocorticoids (n)		HC (4) Pred (2)	Pred (13)	Pred (8)	0.126
Overall hypoglycemia	Number of patients(%)	6(100)	13(100)	8(100)	1.000
	Number/patient/day	0.61(0.40,1.31)	0.57(0.22,1.58)	1.91(0.81,2.39)	0.236
	Duration (min)	85(50.63,114.39)	78.21(45.00,132.95)	143(62.25,316.62)	0.256

Level 1 hypoglycemia	Number of patients(%)	6(100)	13(100)	8(100)	1.000
	Number/patient/day	0.61(0.34,1.31)	0.50(0.18,1.54)	1.88(0.81,2.39)	0.194
	Duration (min)	68.25(50.63,105.74)	66.43(53.75,135.34)	116.39(57.03,156.35)	0.629
Level 2 hypoglycemia	Number of patients(%)	5(83.3)	8(61.5)	6(75.0)	0.560
	Number/patient/day	0.11(0.05,0.36)	0.07(0.00,0.61)	0.54(0.07,1.44)	0.300
	Duration (min)	37.50(22.50,72.19)	30(0.00,60.05)	86.77(10.31,233.36)	0.220
Extended hypoglycemia	Number of patients(%)	3(50.0)	7(53.8)	6(75.0)	0.538
	Number/patient/day	0.07(0.00,0.38)	0.07(0.00,0.44)	0.65(0.11,1.37)	0.193
Extended Level 2 hypoglycemia	Number of patients(%)	2(33.3)	3(23.1)	5(62.5)	0.234
	Number/patient/day	0.00(0.00,0.07)	0.00(0.00,0.04)	0.07(0.00,0.07)	0.552
Mean monitoring days		13.88(13.80,13.90)	13.70(13.04,13.90)	13.18(12.80,13.80)	0.163

Mean glucose (mmol/L)	24h period	5.74±0.91	5.97±0.81	5.06±1.45	0.166
	Nocturnal	4.72±0.38	4.58±0.71	4.07±1.20	0.292
	Diurnal	6.00±1.08	6.40±0.88	5.35±1.54	0.150
	Breakfast period	4.73(4.19,5.64)	6.10(5.21,6.88)	5.11(4.00,5.87)	<0.001
	Lunch period	6.38(5.59,7.73)	7.33(6.33,8.19)	5.80(4.94,7.64)	<0.001
	Dinner period	6.55(5.72,8.07)	6.51(5.63,7.48)	5.55(4.53,7.13)	<0.001
TIR (3.9-7.8 mmol/L) %)	24h period	80.83±12.24	71.92±15.02	61.38±21.70	0.115
	Nocturnal	94.50(79.75,96.50)	84.00(62.00,92.00)	43.00(13.75,76.25)	0.017
	Diurnal	78.33±15.90	71.69±14.15	66.75±22.45	0.475
TAR (≥7.8 mmol/L) (%)	24h period	5.00(2.75,21.25)	16.00(7.50,22.00)	1.00(0.25,10.25)	0.051
	Nocturnal	0.00(0.00,0.50)	1.00(0.00,1.50)	0.00(0.00,0.00)	0.114
	Diurnal	6.50(3.50,28.75)	21.00(10.00,29.00)	1.50(0.25,13.25)	0.055
	Breakfast period	0.00(0.00,11.11)	0.00(0.00,25.00)	0.00(0.00,0.00)	<0.001
	Lunch period	0.00(0.00,44.44	37.50(5.56,62.50)	0.00(0.00,38.12)	<0.001
	Dinner period	11.11(0.00,55.56)	11.11(0.00,37.50)	0.00(0.00,33.33)	<0.01
TBR (<3.9 mmol/L) (%)	24h period	3.50(1.75,10.75)	3.00(1.50,15.00)	20.50(5.00,52.00)	0.175
	Nocturnal	3.50(2.75,16.50)	10.00(4.00,30.00)	48.50(9.75,82.50)	0.134
	Diurnal	2.00(1.00,10.25)	1.00(0.00,10.00)	13.00(2.25,41.75)	0.178
	Breakfast period	0.00(0.00,18.25)	0.00(0.00,0.00)	0.00(0.00,39.24)	<0.001
	Lunch period	0.00(0.00,0.00)	0.00(0.00,0.00)	0.00(0.00,0.00)	<0.001

	Dinner period	0.00(0.00,0.00)	0.00(0.00,0.00)	0.00(0.00,12.50)	<0.001
TBR (<3 mmol/L) (%)	24h period	0.00(0.00,2.25)	0.00(0.00,3.00)	4.00(0.25,24.5)	0.078
	Nocturnal	0.50(0.00,2.50)	1.00(0.00,7.00)	9.50(1.00,38.25)	0.128
	Diurnal	0.00(0.00,2.25)	0.00(0.00,1.00)	0.50(0.00,20.25)	0.453
CV (%)	24h period	27.17±7.19	29.08±7.53	29.00±6.19	0.849
	Nocturnal	9.33±3.20	10.62±2.79	10.33±3.05	0.678
	Diurnal	23.50(18.00,29.25)	23.00(19.00,28.00)	24.00(19.00,27.75)	0.999
	Breakfast period	11.39(6.98,22.80)	13.35(7.95,19.40)	13.50(6.64,18.30)	0.431
	Lunch period	12.28(8.09,16.50)	14.42(9.41,22.86)	12.73(8.06,20.74)	<0.01
	Dinner period	11.74(8.29,18.24)	13.46(8.05,20.83)	12.70(6.57,21.54)	0.606
SD (mmol/L)	24h period	1.14(1.09,1.31)	1.39(1.17,1.66)	1.10(1.04,1.24)	0.235
	Nocturnal	0.42(0.29,0.54)	0.49(0.39,0.60)	0.37(0.27,0.57)	0.479
	Diurnal	1.25(0.96,2.03)	1.46(1.25,1.85)	0.99(0.92,1.48)	0.187
	Breakfast period	0.49(0.30,1.38)	0.80(0.46,1.22)	0.65(0.34,0.99)	<0.001
	Lunch period	0.79(0.47,1.18)	0.99(0.69,1.61)	0.82(0.46,1.25)	<0.001
	Dinner period	0.76(0.51,1.35)	0.89(0.51,1.33)	0.73(0.34,1.26)	0.118
MAGE (mmol/L)	24h period	2.39(2.22,3.28)	3.10(2.79,3.95)	2.52(2.29,2.87)	0.281
	Nocturnal	1.14(0.87,1.57)	1.27(0.98,1.72)	1.09(0.85,1.50)	0.652
	Diurnal	4.02±2.06	4.25±1.33	3.32±1.46	0.416
	Breakfast period	1.50(0.70,3.45)	2.30(1.10,3.50)	1.70(0.88,2.50)	<0.01
	Lunch period	2.20(1.18,3.10)	2.50(1.60,4.05)	2.00(1.10,3.10)	<0.001

	Dinner period	1.95(1.30,3.13)	2.30(1.30,3.40)	1.80(0.80,3.55)	0.161
MODD	24h period	0.79(0.78,0.87)	0.91(0.78,0.99)	0.66(0.46,0.84)	0.311
(mmol/L)	Nocturnal	0.54(0.42,0.65)	0.59(0.48,0.67)	0.50(0.29,0.85)	0.595
	Diurnal	0.97(0.83,1.69)	1.15(1.02,1.42)	0.93(0.77,1.36)	0.250
	Breakfast period	0.68(0.40,1.30)	0.97(0.61,1.41)	0.63(0.40,1.09)	<0.001
	Lunch period	1.11(0.74,2.17)	1.27(0.88,1.92)	1.15(0.64,1.99)	0.592
	Dinner period	1.40(0.95,2.34)	1.13(0.69,1.59)	1.01(0.57,2.16)	<0.01

BMI, body mass index; Pred, prednisone; HC, hydrocortisone; CV, coefficient of variability; SD, the standard deviation of mean glucose; MAGE, mean amplitude of glycemic excursions; MODD, mean of daily differences of glucose; TBR, time below range, glucose < 3.9 mmol/L and < 3.0 mmol/L; TIR, time in range, glucose within 3.9-7.8 mmol/L; TAR, time above range, glucose ≥7.8 mmol/L; 24h period, 0:00 am-11:59 pm; Diurnal ,6:00 am to 11:59 pm; Nocturnal, 0:00 am to 6:00 am; Breakfast period, 8:00 am-10:00 am; Lunch period,12:00 pm-14:00 pm; Dinner period, 18:00 pm-20:00 pm;

Supplementary Table 3. Comparison of clinical characteristics and CGM parameters in patient group classified by types of glucocorticoids.

parameters	HC	Pred	p-Value
N	6	21	
Age (yrs)	62.67±9.54	59.14±8.27	0.381
Male, n (%)	5 (83)	10 (48)	0.182

BMI (kg/m²)		20.23±2.86	23.57±4.21	0.081
Disease duration (month)		11.0(4.0,126.0)	4.5(0.8,23.2)	0.598
Daily glucocorticoid dosage[a] (mg)		20.00(19.38,21.25)	5.00(3.75,7.50)	0.950
Administration Frequency (n)	Once per day	3	12	
	Twice per day	3	9	1.000
Mean monitoring days		13.90(13.75,13.90)	13.90(13.75,13.90)	0.711
Overall hypoglycemia	Number of patients(%)	6(100)	21(100)	1.000
	Number/patient/day	0.32(0.14,1.34)	0.86(0.43,2.09)	0.102
	Duration (min)	45.00(45.00,95.13)	105(59.66,159.41)	0.061
Level 1 hypoglycemia	Number of patients(%)	6(100)	21(100)	1.000
	Number/patient/day	0.29(0.14,1.27)	0.86(0.40,2.09)	0.109
	Duration (min)	46.25(45.00,80.98)	93.13(66.21,134.30)	0.038
Level 2 hypoglycemia	Number of patients(%)	3(50.0)	16(76.2)	0.319
	Number/patient/day	0.04(0.00,0.49)	0.22(0.04,0.64)	0.261

	Duration (min)	15(0.00,51.31)	45(15,97.08)	0.124
Extended hypoglycemia	Number of patients(%)	2(33.3)	14(66.7)	0.187
	Number/patient/day	0(0.00,0.26)	0.00(0.14,0.90)	0.116
Extended Level 2 hypoglycemia	Number of patients(%)	1(16.7)	9(42.9)	0.363
	Number/patient/day	0.00(0.00,0.12)	0.00(0.00,0.07)	0.343
Mean glucose (mmol/L)	24h period	6.34±1.22	5.46±1.00	0.080
	Nocturnal	5.11±0.95	4.27±0.74	0.030
	Diurnal	6.70±1.31	5.80±1.11	0.104
	Breakfast period	6.28(5.08,7.40)	5.29(4.49,6.21)	<0.001
	Lunch period	7.21(6.14,8.16)	6.57(5.51,7.98)	<0.01
	Dinner period	6.04(5.47,7.22)	6.43(5.31,7.54)	0.925
TIR (3.9-7.8mmol/L) (%)	24h period	74.00(54.25,86.00)	76.00(61.50,85.50)	0.977
	Nocturnal	81.50(70.00,95.00)	82.00(28.00,92.50)	0.521
	Diurnal	75.00(45.25,85.00)	75.00(61.50,85.50)	0.748
TAR (≥ 7.8mmol/L) (%)	24h period	15.50(3.75,44.25)	8.00(1.50,17.50)	0.161
	Nocturnal	0.50(0.00,16.00)	0.00(0.00,1.00)	0.279
	Diurnal	20.00(4.75,53.75)	11.00(2.00,23.50)	0.170
	Breakfast period	0.00(0.00,50.00)	0.00(0.00,11.11)	<0.001

	Lunch period	33.33(0.00,63.54)	12.50(0.00,50.00)	0.016
	Dinner period	0.00(0.00,31.25)	0.00(0.00,44.44)	0.597
TBR	24h period	1.5(0.75,10.5)	5.00(2.00,27.00)	0.084
(<3.9mmol	Nocturnal	4.00(1.00,21.25)	12.00(5.00,66.50)	0.080
/L) (%)	Diurnal	0.00(0.00,6.50)	3.00(1.00,15.50)	0.058
	Breakfast period	0.00(0.00,0.00)	0.00(0.00,14.29)	0.029
	Lunch period	0.00(0.00,0.00)	0.00(0.00,0.00)	<0.001
	Dinner period	0.00(0.00,0.00)	0.00(0.00,0.00)	0.091
TBR	24h period	0.00(0.00,1.75)	1.00(0.00,4.50)	0.285
(<3mmol/L	Nocturnal	0.50(0.00,5.00)	1.00(0.00,13.00)	0.321
) (%)	Diurnal	0.00(0.00,0.75)	0.00(0.00,2.00)	0.312
CV (%)	24h period	28(24.25,30.25)	26(23,34.50)	0.930
	Nocturnal	12.17±4.67	9.81±2.32	0.279
	Diurnal	24.00±4.94	24.10±6.79	0.975
	Breakfast period	16.33(12.60,20.46)	11.46(6.68,18.70)	<0.001
	Lunch period	12.94(8.25,19.76)	13.57(9.00,19.32)	0.573
	Dinner period	12.09(7.67,17.81)	12.93(7.88,22.11)	0.271
SD	24h period	1.62(1.30,2.32)	1.39(1.20,1.75)	0.397
(mmol/L)	Nocturnal	0.66±0.34	0.43±0.14	0.164
	Diurnal	1.46(1.25,2.11)	1.28(0.98,1.67)	0.232
	Breakfast period	1.00(0.72,1.34)	0.62(0.34,1.03)	<0.001
	Lunch period	0.86(0.63,1.53)	0.89(0.56,1.30)	0.450

	Dinner period	0.74(0.47,1.23)	0.78(0.48,1.35)	0.590
MAGE	24h period	4.17(2.90,5.79)	3.36(2.79,4.52)	0.414
(mmol/L)	Nocturnal	1.91±1.05	1.21±0.34	0.167
	Diurnal	4.10(3.03,5.69)	3.54(2.54,4.56)	0.466
	Breakfast period	2.80(1.75,3.55)	1.60(0.80,2.70)	<0.001
	Lunch period	2.20(1.50,3.90)	2.20(1.20,3.50)	0.275
	Dinner period	2.00(1.20,3.10)	2.00(1.10,3.45)	0.723
MODD	24h period	0.94(0.84,1.65)	0.98(0.81,1.20)	0.662
(mmol/L)	Nocturnal	0.85±0.55	0.54±0.17	0.226
	Diurnal	1.06(0.94,1.63)	1.09(0.87,1.46)	0.748
	Breakfast period	0.89(0.59,1.50)	0.79(0.48,1.35)	0.042
	Lunch period	1.24(0.90,1.94)	1.26(0.74,2.00)	0.526
	Dinner period	1.12(0.54,1.65)	1.23(0.74,1.97)	0.067

[a] Daily glucocorticoid dosage was converted to Pred equivalent (mg).

BMI, body mass index; Pred, prednisone; HC, hydrocortisone; CV, coefficient of variability; SD, the standard deviation of mean glucose; MAGE, mean amplitude of glycemic excursions; MODD, mean of daily difference of glucose; TBR, time below range, glucose <3.9 mmol/L and glucose <3.0 mmol/L; TIR, time in range, glucose within 3.9-7.8 mmol/L; TAR, time above range, glucose ≥7.8 mmol/L; 24h period, 0:00 am-11:59 pm; Diurnal ,6:00 am to 11:59 pm; Nocturnal, 0:00 am to 6:00 am; Breakfast period, 8:00am-10:00am; Lunch period,12:00 pm-14:00 pm; Dinner period, 18:00 pm-20:00 pm;

Supplementary Table 4. Comparison of the clinical characteristics and CGM parameters between ICIs-induced adrenal insufficiency and the other etiology of the patient group.

parameters		ICIs-induced group	non-ICIs-induced group	p-Value
N		11	16	
Age (yrs)		62(54,66)	60(55,68)	0.961
Male, n (%)		7(64)	8(50)	0.492
BMI (kg/m^2)		21.59±3.47	23.68±4.46	0.204
Disease duration (month)		11.0(2.0,30.0)	7.0(2.3,58.5)	0.882
Daily glucocorticoid dosage (based on Pred, mg)		5.00(5.00,6.25)	5.00(3.13,7.50)	0.674
Types of glucocorticoids(n)		Pred (7) HC (4)	Pred (14) HC (2)	0.187
Overall hypoglycemia	Number of patients(%)	11(100)	16(100)	1.000
	Number/patient/day	0.50(0.15,2.31)	0.93(0.47,1.96)	0.537
	Duration (min)	100.91(45.00,161.00)	86.88(58.59,140.68)	0.921
Level 1 hypoglycemia	Number of patients(%)	11(100)	16(100)	1.000
	Number/patient/day	0.43(0.15,2.31)	0.93(0.45,1.94)	0.639
	Duration (min)	95.45(45.00,148.26)	72.00(62.30,116.77)	0.961

Level 2 hypoglycemia	Number of patients(%)	8(72.7)	11(68.6)	1.000
	Number/patient/day	0.07(0.00,0.93)	0.25(0.00,0.56)	0.822
	Duration (min)	30.00(0.00,68.07)	46.50(0.00,92.34)	0.634
Extended hypoglycemia	Number of patients(%)	6(54.5)	10(62.5)	0.710
	Number/patient/day	0.08(0.00,1.01)	0.08(0.00,0.72)	0.878
Extended Level 2 hypoglycemia	Number of patients(%)	3(27.3)	7(43.8)	0.448
	Number/patient/day	0.00(0.00,0.07)	0.00(0.00,0.07)	0.390
Mean monitoring days		13.92(13.67,13.92)	13.92(13.80,13.92)	0.760
Mean glucose (mmol/L)	24h period	5.8±1.28	5.50±0.95	0.388
	Nocturnal	4.53±1.08	4.41±0.68	0.729
	Diurnal	6.27±1.38	5.81±1.05	0.326
	Breakfast period	5.94(4.69,7.03)	5.28(4.54,6.19)	<0.001
	Lunch period	6.91(5.76,8.17)	6.55(5.61,7.99)	0.159
	Dinner period	6.54(5.37,8.08)	6.24(5.39,7.21)	0.025
TIR (3.9-7.8mm	24h period	60.57(51.53,81.74)	78.66(69.51,85.35)	0.126
	Nocturnal	79.46(11.01,94.05)	82.99(53.13,93.30)	0.748

ol/L) (%)	Diurnal	68.30(46.16,77.39)	81.66(72.31,85.61)	0.114
TAR (≥	24h period	15.78(4.04,36.15)	7.85(1.34,17.04)	0.183
7.8mmol/L)	Nocturnal	0.32(0.00,1.49)	0.00(0.00,1.04)	0.329
(%)	Diurnal	20.60(5.43,47.96)	10.24(1.62,22.31)	0.183
	Breakfast period	0.00(0.00,39.24)	0.00(0.00,0.00)	<0.001
	Lunch period	33.33(0.00,55.56)	12.50(0.00,0.50)	0.159
	Dinner period	11.11(0.00,50.00)	0.00(0.00,37.50)	<0.01
TBR	24h period	2.39(0.61,36.51)	5.38(2.12,20.10)	0.622
(<3.9mmol/	Nocturnal	6.25(1.79,85.12)	12.93(3.87,36.24)	0.748
L) (%)	Diurnal	1.10(0.50,20.10)	2.30(1.02,13.20)	0.693
	Breakfast period	0.00(0.00,11.11)	0.00(0.00,8.33)	0.930
	Lunch period	0.00(0.00,0.00)	0.00(0.00,0.00)	0.765
	Dinner period	0.00(0.00,0.00)	0.00(0.00,0.00)	0.577
TBR	24h period	0.23(0.00,4.51)	1.08(0.08,3.09)	0.804
(<3mmol/L)	Nocturnal	0.89(0.00,13.10)	2.38(0.30,6.40)	0.823
(%)	Diurnal	0.00(0.00,1.50)	0.25(0.00,2.50)	0.656
CV (%)	24h period	33.42(27.63,35.25)	25.51(22.14,27.70)	0.061
	Nocturnal	10.39±3.58	10.31±2.86	0.948
	Diurnal	26.68±7.83	22.29±4.56	0.077
	Breakfast period	16.37(11.15,24.31)	11.08(6.33,16.72)	<0.001
	Lunch period	14.98(8.98,23.07)	12.51(8.68,18.61)	0.020
	Dinner period	14.38(8.69,23.04)	11.58(7.20,18.54)	<0.01

SD	24h period	1.76(1.28,2.51)	1.38(1.20,1.48)	0.076
(mmol/L)	Nocturnal	0.42(0.28,0.51)	0.46(0.31,0.59)	0.693
	Diurnal	1.54(1.02,2.27)	1.25(0.97,1.40)	0.054
	Breakfast period	1(0.54,1.54)	0.57(0.32,0.92)	<0.001
	Lunch period	1.06(0.56,1.6)	0.82(0.56,1.26)	<0.01
	Dinner period	0.92(0.52,1.70)	0.71(0.43,1.19)	<0.01
MAGE	24h period	4.69(2.91,5.93)	3.20(2.77,3.86)	0.068
(mmol/L)	Nocturnal	1.24(0.86,1.58)	1.20(0.97,1.59)	0.961
	Diurnal	4.76(2.66,5.98)	3.31(2.51,3.93)	0.076
	Breakfast period	2.50(1.45,4.00)	1.40(0.75,2.40)	<0.001
	Lunch period	2.70(1.50,4.20)	2.00(1.20,3.20)	<0.01
	Dinner period	2.40(1.30,4.60)	1.80(1.00,3.00)	<0.001
MODD	24h period	1.17(0.89,1.44)	0.88(0.80,0.99)	0.043
(mmol/L)	Nocturnal	0.57(0.37,0.65)	0.58(0.43,0.68)	0.961
	Diurnal	1.36(0.99,1.50)	1.00(0.86,1.15)	0.068
	Breakfast period	1.05(0.625,1.69)	0.68(0.44,1.11)	<0.001
	Lunch period	1.39(0.84,2.15)	1.18(0.73,1.83)	0.058
	Dinner period	1.34(0.69,2.36)	1.13(0.68,1.63)	0.016

ICIs, immune checkpoint inhibitors; BMI, body mass index; Pred, prednisone; HC, hydrocortisone; CV, coefficient of variability; SD, the standard deviation of mean glucose; MAGE, mean amplitude of glycemic excursions; MODD, mean of daily differences of glucose; TBR, time below range, glucose <3.9 mmol/L and <3.0 mmol/L;

TIR, time in range, glucose within 3.9-7.8 mmol/L; TAR, time above range, glucose ≥ 7.8 mmol/L; 24h period, 0:00 am-11:59 pm; Diurnal ,6:00 am to 11:59 pm; Nocturnal, 0:00 am to 6:00 am; Breakfast period, 8:00 am-10:00 am; Lunch period, 12:00 pm-14:00 pm; Dinner period, 18:00 pm-20:00 pm;
